# Highly conformable chip-in-foil implants for neural applications

**DOI:** 10.1038/s41378-023-00527-x

**Published:** 2023-05-09

**Authors:** Thomas Stieglitz, Calogero Gueli, Julien Martens, Niklas Floto, Max Eickenscheidt, Markus Sporer, Maurits Ortmanns

**Affiliations:** 1 Laboratory for Biomedical Microtechnology, Department of Microsystems Engineering - IMTEK, University of Freiburg, D-79110 Freiburg, Germany; 2 BrainLinks-BrainTools// IMBIT, University of Freiburg, D-79110 Freiburg, Germany; 3 Institute of Microelectronics, University of Ulm, D-89081 Ulm, Germany

**Keywords:** Chip-in-Foil, Conformable Substrate, Microfabrication, Hybrid Implants, Bioelectronic Devices, Polyimide, Implantable Flexible System, Electrical and electronic engineering, Electronic devices

## Abstract

Demands for neural interfaces around functionality, high spatial resolution, and longevity have recently increased. These requirements can be met with sophisticated silicon-based integrated circuits. Embedding miniaturized dice in flexible polymer substrates significantly improves their adaptation to the mechanical environment in the body, thus improving the systems’ structural biocompatibility and ability to cover larger areas of the brain. This work addresses the main challenges in developing a hybrid chip-in-foil neural implant. Assessments considered (1) the mechanical compliance to the recipient tissue that allows a long-term application and (2) the suitable design that allows the implant’s scaling and modular adaptation of chip arrangement. Finite element model studies were performed to identify design rules regarding die geometry, interconnect routing, and positions for contact pads on dice. Providing edge fillets in the die base shape proved an effective measure to improve die-substrate integrity and increase the area available for contact pads. Furthermore, routing of interconnects in the immediate vicinity of die corners should be avoided, as the substrate in these areas is prone to mechanical stress concentration. Contact pads on dice should be placed with a clearance from the die rim to avoid delamination when the implant conforms to a curvilinear body. A microfabrication process was developed to transfer, align, and electrically interconnect multiple dice into conformable polyimide-based substrates. The process enabled arbitrary die shape and size over independent target positions on the conformable substrate based on the die position on the fabrication wafer.

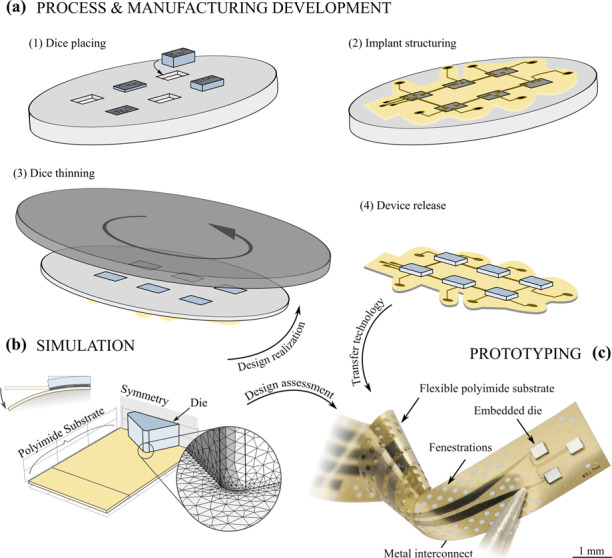

## Introduction

Trends in neurotechnology today are moving toward mechanically compliant, miniaturized implants with high integration density. To avoid additional implantation needed for the replacement of such systems, lifetimes can target several decades. A large number of neural implants, such as brain pacemakers^1,2^, retina implants^3^, or brain–computer interfaces^4,5^, are the subject of research or are being used in rehabilitation medicine to restore lost body functions, promote health, and improve the quality of life of patients by providing a link between the human nervous system and technological systems^6–8^. Better understanding and cross-curricular work in the fields of neuroscience, biotechnology, material sciences, and related engineering disciplines can pave the way for advancements in such systems.

A high level of complexity can be realized by sophisticated silicon (Si)-based integrated circuits (ICs)^9–11^. High integration density enables high spatial resolution at the cost that only small areas of the brain can be covered. However, mechanically stiff Si induces a foreign body response leading to tissue encapsulation of implants and insulation of electrode contacts from neurons, which, in return, compromises functionality in chronic studies and applications^12,13^. It has been shown that this effect can be minimized by matching the implant’s mechanical properties to the host tissue and making the implant conformable to adapt to the biological surface shape. Conformability leads to intimate contact and, therefore, high efficacy and minimal invasiveness^14^. Polyimides (PIs) exhibit excellent chemical stability and biocompatibility, desirable mechanical properties, low water uptake, reduced plasticization, and high electric resistivity and dielectric strength; they are, therefore, among the most commonly used substrate materials for new-generation mechanically compliant implants^15–18^. They are excellent substrate materials for flexible neural implants because they are able to conform to curvilinear structures such as the brain^19^, are compatible with microfabrication processes, and can be manufactured in sub-micrometer thickness; their high performance and long-term stability have been shown in several studies^20–26^. Therefore, the combination of mechanically compliant PI-based substrates and small Si-based circuits was proposed to develop bioelectronic devices with complex functionality and good adaptation to the biomechanics of the host tissue^27,28^ that are able to cover larger brain areas. Intrinsically flexible electronics have been made possible with organic transistors and circuits, and improvements in stability, device performance, cost, and fabrication methods have been made in recent years. Utilizing such materials could meet requirements concerning mechanical flexibility; however, they currently do not offer the integration density and complexity possible with mature and reliable Si-based technology^29–31^.

Anatomical boundary conditions in terms of available space and desired electrode count or location for recording or stimulation of neural signals may vary between target regions, species, or even individuals^32,33^. Thus, a modular arrangement of circuit components can be beneficial by enabling fast adaptation of the implant design to address a large number of established and new fields of application. These comprise monitoring of large cortical areas, organ healing processes, or bridging functions in the peripheral nervous system—highly specialized prototypes for diverse scientific questions and medical treatments.

An implant should always be designed considering the mechanical and geometrical conditions of the target tissue^34^, such as the softness of the brain in general and the curvature of the brain region of interest. The radius of curvature in the sensorimotor cortex of a rat, for example, can range from several hundred microns to a few millimeters^32^, while radii on the cortex of a human brain are much larger. Conformability is the ability of a thin membrane or foil to attach to a body and take on its shape^35^, and due to better conformability, closer contact between electrodes in the implant and the cortex can be achieved^6^. In addition to foil thickness, conformability depends on many other factors: mechanical properties, geometries of the implant and the cortex, surface tension of the wetting liquid^36^, and the biological fluid between the brain and implant. Therefore, the geometry, size, and stiffness of implants should be tailored to the specific application to reduce the biotic/abiotic gap and achieve recordings with a high spatial resolution^37^. Hybrid bioelectronic devices, consisting of sophisticated ICs modularly distributed on miniaturized flexible polymeric substrates, are a promising approach to overcome a central challenge in neural implants: the combination of complex functionality at high channel density and longevity in the harsh biological environment. The virtue of the proposed method is the possibility of scaling down the size of Si-based dice and distributing tasks between dice connected by thin-film interconnect lines on the flexible substrate. This is accompanied by a lower bending stiffness compared to a single but larger die and good adaptability of the technical system in terms of the range of function, substrate dimensions, and target anatomy. This work formulates design rules regarding implant geometries and thin-film routing with the background question of the existence of critical points for mechanical stress in the implant. Questions concerning system integrity and good electrode-tissue contact are essentially probed through the scope of developing a chronic implant. For example, contact pads on dice should be positioned in areas without critical stress/delamination due to the substrate conformation to the curvilinear brain surface. Reliable implant function can be achieved if no delamination of dice from the substrate or interconnect damage takes place.

## Results

**Fig. 1:**
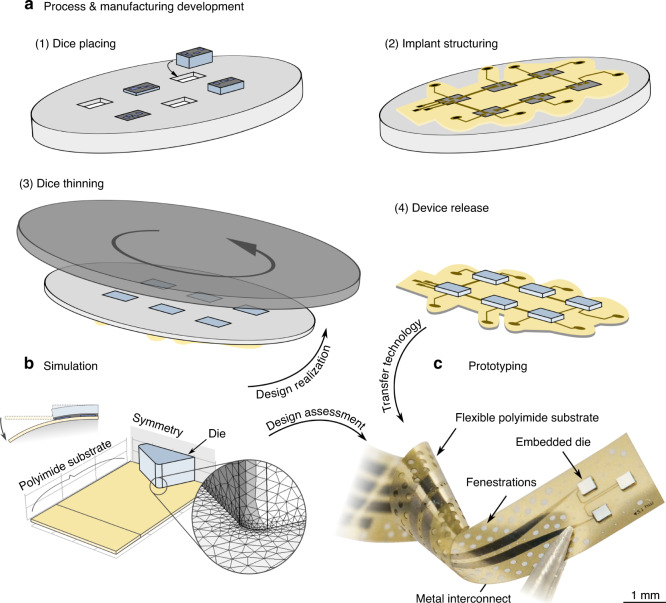
**Design assessment and transfer technology for the development of a chip-in-foil implant. a** Simplified sketch of the general concept for batch transfer of multiple silicon-based dice into flexible polyimide-based substrates by (1) placement of dice into a carrier substrate, (2) structuring of the flexible polyimide substrate, metal interconnect lines, contact pads, and electrode sites, (3) thinning of the die backside to the desired thickness, and (4) release of the devices, as simulated by **b** a finite element model of the mechanical interaction at the die–substrate interface under implant conformation to a curvilinear body to optimize die design. **c** Photograph of a flexible PI-based device prototype with three Si-based dice (each 390 × 390 µm² and 24 µm thin) connected in a chain by sputter-deposited metal interconnects

A finite element model (FEM) simulation was developed to describe the mechanical behavior of the hybrid implant when it conformed to a brain surface with the aim of design optimization. The brain area considered in this study was the somatosensory cortex of rats, approximated as a cylinder with a 3 mm radius of curvature^32^. Furthermore, the development of a transfer process for Si-based dice into flexible PI-based substrates and their three-dimensional placement accuracy was the subject of investigation. The results were combined to manufacture prototype implants (Fig. 1).

### Design assessment: implant geometry

**Fig. 2:**
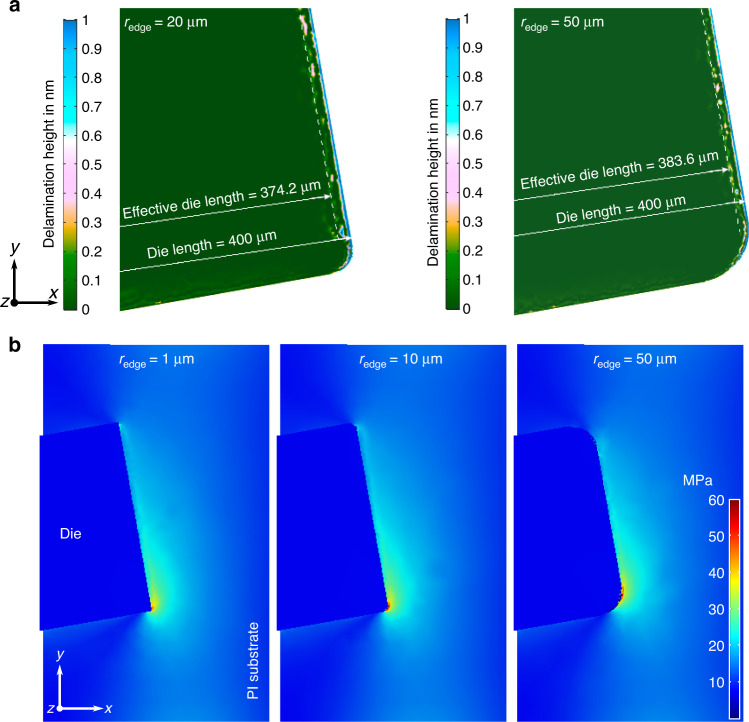
**Overview of the finite element model results. a** Delamination height at the interface between the die and polyimide substrate with effective die length represented with a color coding scale. The die was rotated by an angle *α*_Z_ of 10°, and the edge radius fillet was 20 and 50 µm (die length *l*_die_ = 400 µm, quadratic base shape). The polyimide substrate around the die was excluded from the plot for better visibility. **b** Mechanical stress distribution in the polyimide (PI) substrate caused by a die of 400 µm length, rotated by an angle *α*_Z_ of 10° and deformation onto a cylindrical surface for edge fillet radii varying between 1 and 50 µm

The mechanical stress at the die–substrate interface concerning the variation in edge fillet radius was determined. The maximum delamination height between the flexible PI substrate (thickness of 10 µm) and the die (thickness of 100 µm) was taken as an indicator of how large the effect due to the filleted die corners on the total delamination is. The possible deformation of the PI substrate increases with delamination. Therefore, a more considerable maximum delamination height can be associated with a larger delaminated area. With larger edge filleting (and lower maximum delamination), a lower distance between the delamination edge and the die edge was observed (in the *XY*-plane). The effective area for a die varied with the fillet radius (Fig. 2a). Most delamination (>1 nm) was observed at the die edge. However, delamination could be found further toward the die center. Maximum delamination of 24 nm occurred along the die edge with *r*_edge_ = 20 µm at a distance of up to 12.8 µm toward the die center or 14.1 nm at 8.2 µm for *r*_edge_ = 50 µm. Since even minor delamination can damage the electrical contacts between pads on dice and interconnects in the PI substrate, the largest distance from the die edge to the individual points of delamination was subtracted from the edge length of the die to determine an effective die length. This resulted in a usable area of 87.8% of the total die area for *r*_edge_ = 20 µm and 92.3% for *r*_edge_ = 50 µm. The die with a 50 µm fillet radius has a lower geometrical area (0.158 mm²) compared to the die with a 20 µm radius. However, the effective area of 0.146 mm², caused by rotation around *α*_Z_ along with delamination, is more extensive than for the die with a lower fillet radius. Even larger fillets could lead to a loss of die area that is not compensated by the gain in effective die area, which must be evaluated accordingly. Local stress concentration in the PI substrate occurred at the corners of the die (Fig. 2b) and was distributed along with the edge fillets. The distribution of stress at the corners of the interface was similar in size and magnitude for different radii (between 1 and 50 µm). Based on the simulated stress distribution, conclusions can be drawn for interconnect placement on the PI substrate. Since areas with the most prominent stress were observed in the vicinity of corners for all variations of the fillet radius, interconnect routing should be avoided in this area as much as possible. The assumption of a die misaligned by 10° to the curvatures orthogonal was made to include both process and implantation tolerances to establish design rules for possible use cases. A more considerable effective die length and area could be expected with lower misalignment. The PI substrate was simplified as a solid foil without consideration of thin-film metal interconnects (with approximately one order of magnitude larger Young’s modulus^38^) and substrate fenestrations. The enhanced thickness ratio of metal to PI is 0.03, and with the thickness related to bending stiffness to the third power, it could be expected that the PI substrate thickness is the critical parameter. An additional decrease in bending stiffness by fenestration (opening-to-substrate ratio anticipated between 0.1 and 0.2) can be assumed due to a smaller equivalent width^19^. This approach has been shown to conform better to the brain surface^26^ and positively impact the meningeal tissue response, while solid substrate showed thick tissue formation between tissue and recording electrode sites, which in return can compromise performance^39^. The metal area on dice was neglected (to lower the calculation resources needed) since it only has an influence on adhesion in the area of the pads, which is much smaller than the die area. PI is the first process step, so the subsequent metal layers have no structural influence on the system behavior.

### Transfer technology

**Fig. 3:**
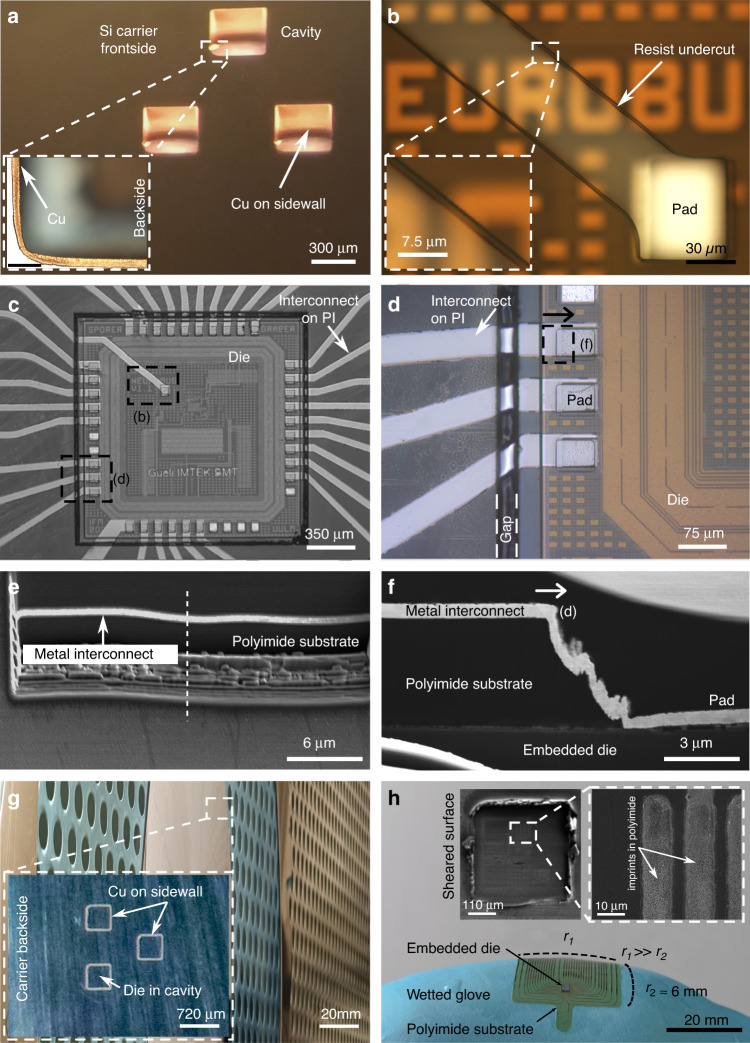
**Transfer process results. a** Transfer carrier frontside with three cavities for dice, sidewalls covered with a copper sacrificial layer. **b** Photoresist for deposition of metal interconnects on the die surface with a sharp undercut. **c** Sputtered metal interconnects after lift-off. **d** Detailed view of the pad and inside gap between the die and carrier with a continuous interconnect line. **e** SEM image of the cross-section of the PI/metal/PI interface. The dashed line indicates the position of the die edge*. **f** SEM image of the cross-section at the interface between the PI and die with the metal interconnect forming a via through the dry-etched PI for die contact*. **g** Backside of carrier on grinding tape with optical micrograph of three dice placed in cavities of the carrier after grinding dice and carrier backside to the desired thickness*. **h** Photograph of a flexible PI-based device with Si-based dice (1620 × 1620 µm²) conforming to the spherical surface of a wetted glove (bending radius *r*_2_ approximately 6 mm). The inset shows an SEM image of the void-free PI surface after mechanical shearing off a die with a close-up view of the imprint topography in the polyimide due to 200-nm-thin metal structures on the die. * Reproduced from^44^ © [2021] IEEE

The transfer of Si-based dice into mechanically flexible PI-based substrates was performed using a carrier equipped with cavities in which dice were placed and subsequent level microfabrication of mechanically conformable substrates directly on the carrier. Galvanic deposition of the sacrificial copper (Cu) layer on the cavity sidewalls for die release at the end of the process sequence was successfully performed (Fig. 3a). Cu (5.81 ± 1.45 µm, *n* = 75, determined with optical micrographs and ImageJ software) was deposited over the entire cavity height down to the carrier backside surface (inset in Fig. 3a). Deposition was intentionally limited to the cavity sidewalls; the dicing tape cover averted deposition on the frontside surface, and no backside deposition occurred due to the bare Si surface. The frontside deposition was not carried out, as the electrodeposited layer had surface roughness orders of magnitude larger than the polished Si wafer^40^, which would be transferred to the subsequent PI and metallization layers. Dry etching of the cavities was performed with an aluminum etch stop to prevent passivation gas deposition on the carrier backside that potentially lowered the adhesion of the backside PI (intended for die fixation during steps 3–7 in Fig. 6). The cavities were designed to be slightly larger than the dice, which ensured uncompromised insertion and tight fit. A substrate backside that had been leveled by mechanical grinding and sufficiently cleaned enabled a well-adhering backside PI, which was essential for subsequent process steps allowing fixation on vacuum chucks during spin coating, mask alignment, or dry etching. Spin coating of negative tone photoresist over the PI-filled gap between dice and carrier was successful, and the photoresist mask for interconnects exhibited sharp undercuts on the surface of dice (Fig. 3b, position marked in Fig. 3c) and in the gap. A relatively thick photoresist (4 µm ma-N-1440) was chosen for patterning the metallization layer to enable interruption-free coating over the height topography between dice and carrier substrate, which is essential for continuous interconnection. According to the simulation results from the design assessment in “Results”, no interconnect lines were routed directly over die corners to circumvent an area of stress concentration in the substrate that could otherwise compromise the function of this particular interconnect. Lithography led to satisfactory results, and PVD was able to reliably form all 35 intended interconnect lines (Fig. 3c). Metal interconnects had a minimum width of 30 µm and were deposited interrupt-free over the gap (between carrier and dice, Fig. 3d, e) and contact pads on dice by sputter deposition of a WTi/Au/WTi stack and subsequent lift-off technique. Metal interconnects were well integrated into PI substrates (Fig. 3e) and formed continuous vias through the dry-etched PI for die contact (Fig. 3f). PI above the contact pads was opened with beveled sidewalls (*α*_Via_ = 61 ± 0.9°, *n* = 5) to facilitate a uniform PVD coating from the PI base layer surface down to the dice contacts. Thinning of dice and carrier backside was performed without displacement or tearing out of dice or impairment of the Cu sacrificial layer between the cavity and dice sidewalls (Fig. 3g). The UV-curable tape was exposed after thinning and removed without any displacement of PI-based samples or damage to the carrier. Large dice on PI substrates with 35 sputtered interconnects were released from the carrier and conformed well to the spherical surface of a wetted glove (Fig. 3h). SEM on the PI surface of dice that were mechanically sheared off after thermally accelerated aging at 60 °C in phosphate-buffered saline showed seamless and void-free integration (Fig. 3h). The close-up view shows the imprint topography in the PI due to metal structures on the die after adhesive failure. No indications of air inclusions in the PI underneath the dice could be observed. As shown in the SEM images, the PI surface is smooth without entrapped voids. This indicates a properly cured polymer, most likely facilitated by allowing one side of the PI layer to exchange with the surrounding atmosphere while annealing. Favorable adhesion between dice and flexible substrate, as well as tight and void-free contact, is desirable to increase the system’s robustness against mechanical stress. The main advantage between the process sequence presented here and other approaches is the embedding of dice into PI before annealing at high temperatures. This eliminated the need for additional adhesive material, such as BCB, which was used in several other approaches as a fixative on already cured PI/polymer substrates^41,42^. With this process, a maximum number of 34 dice were aligned, transferred, and interconnected using a single carrier. Moreover, a larger number of dice can be implemented in this implant design simply by scaling up the number of cavities in Fig. 6(1). Hybrid devices consisting of 10-µm-thick PI-based substrates and three Si-based dice, each 390 × 390 µm² and 24 µm thin (interconnected by sputtered thin-film metal), could be released from the carrier and were highly flexible (Fig. 1c).

In addition to neural recorder ASICs fabricated in CMOS technology and diced by blade dicing, test dice diced by dry etching were transferred into PI-based substrates. Dicing by dry etching allowed for the application of simulation results and the optimized geometry. Test dice featured an embedded thin-film resistor in series to the contact pads for evaluation of the electrical interconnection. Three such dice linked in a chain yield three resistors in series through the contact pads and interconnect lines. The 4-wire measurement of the line resistance for a chain of three dice was 209.1 ± 75.7 Ω (*n* = 5), with the lowest measured value of 115.9 Ω. The ideal value, computed with the structural dimensions and specific resistance of the materials, was 57.1 Ω for a chain of three dice. This deviation potentially resulted from the line resistances combined with an increased contact resistance at the interface to the Al pads on dice, where an oxide layer with increased resistance could have formed. However, in a future study, such contact resistance could be avoided by choosing a different metal as the test structure, which would enable a low-resistance contact.

Two die designs were transferred into PI substrates in this study since approaches with different objectives were pursued in parallel. On the one hand, the optimization was advanced and supported by simulations, for which test dice were built. On the other hand, the embedding of a functional prototype was intended to study and test the electrical connections. Preliminary studies have been performed, and those results have been published elsewhere^43^.

### Accuracy assessment: die placement

**Fig. 4:**
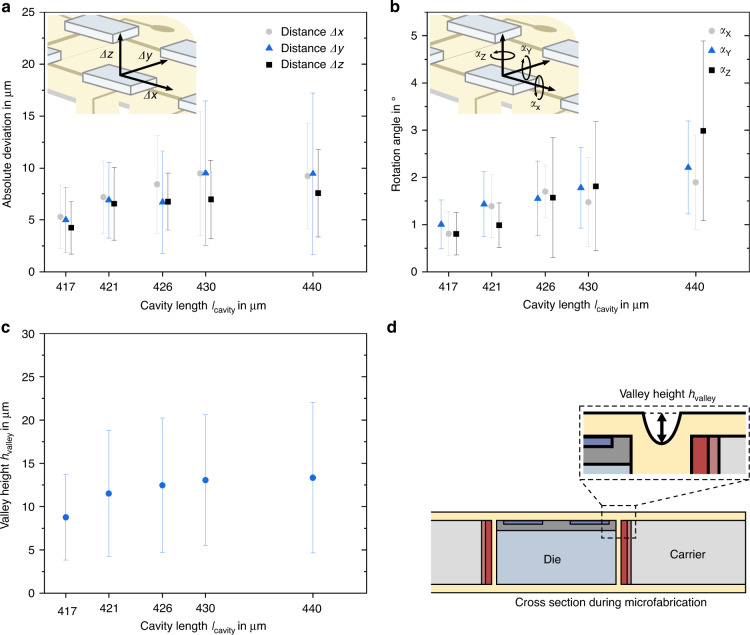
**Overview of the placement accuracy. a** Absolute deviation of dice (390 × 390 µm²) from the ideal position *Δx*, *Δy*, and *Δz* in the *X*-, *Y*- and *Z*-directions and **b** angular rotation error *α*_X_, *α*_Y_, and *α*_Z_ around the *X*-, *Y*- and *Z*-axes for variation in the cavity length. **c** Valley height *h*_valley_ between the PI at the substrate level and inside the gap between the transfer wafer and die. **d** Complementary information for clarification of the parameter location in (**c**). All plots show the mean and standard deviation and the number of samples for cavity lengths: 417 µm (*n* = 45), 421 µm (*n* = 27), 426 µm (*n* = 21), 430 µm (*n* = 15), and 440 µm (*n* = 21)

Deviation from the ideal die position in the *X*-, *Y*- and *Z*-directions was determined for variation of the cavity length in the transfer carrier (Fig. 4a). Average deviations were lowest for the smallest cavity and tended to increase with cavity length, with the lowest mean in-plane translation for the 417-µm cavity of 5.13 ± 3.06 µm (*n* = 90) and a deviation in the *Z*-direction of 4.26 ± 2.53 µm (*n* = 45). A similar trend was observed for die rotation (Fig. 4b), with a mean in-plane rotation of 0.80 ± 0.49° (*n* = 45), a mean out-of-plane rotation of 0.90 ± 0.50° (*n* = 90), and a valley height *h*_valley_ between PI at the substrate level and inside the gap between the transfer carrier and die (Fig. 4c) of 8.75 ± 4.93 µm (*n* = 180). The critical rotation angle *α*_Z,max_ for this case, was 8.97°, according to Eq. (2), with a critical translation of 24.8 µm. The rotational misalignment around the *X*- and *Y*-axes was >0° (Fig. 4b), possibly due to the carrier bow and the not perfectly flat surface during the insertion of dice. If both were perfectly flat and parallel, out-of-plane die rotation would have been prevented. For the smallest cavity of 417 µm length, *h*_valley_ was significantly lower (0.05 level) than for the other cavity lengths. From analysis of the positioning parameters, a trend toward a cavity size of 417 µm for the lowest misalignment could be drawn. Critical rotation and translation were prevented by the geometrical design of dice- and cavity length and critical distance (in this case, between the edge of the contact pad opening and the adjacent interconnect line). Optimization of these parameters led to the maximum possible values below critical rotation and translation. With increasing distance *r*_pad_ of pads from the die center, the risk of misalignment becomes more prominent. Therefore, well-suited cavities will push the capable limits toward smaller pad sizes and pitches and thus the function density of the embedded ICs.

## Discussion

A measure to improve die integrity on the flexible PI-based substrate and increase the area available for contact pads is to provide radius fillets in the design of the die base shape. Maximum delamination of 24 nm occurred along the die edge with *r*_edge_ = 20 µm at a distance of up to 12.8 µm toward the die center or 14.1 nm at 8.2 µm for *r*_edge_ = 50 µm, which resulted in a usable area of 87.8% of the total die area for *r*_edge_ = 20 µm and 92.3% for *r*_edge_ = 50 µm. Further adaptation in the die shape could be made according to the anatomical boundary conditions of a specific application. Since the flexible substrate in the immediate vicinity of die corners is prone to mechanical stress concentration, interconnect lines should not be routed in these areas. Additionally, contact pads on dice should be placed with a clearance from the die rim since the substrate was more affected by delamination in this area when the implant conformed to a curvilinear body. This clearance depends on the radii of curvature of the target region and die base shape. In the exemplary study provided here (for a 400 by 400 µm die on a cylindrical surface with a radius of curvature of 3 mm), a clearance of 10 µm was suggested.

The developed process was based on the placement of individual dice into cavities of a carrier wafer that allowed for subsequent level microfabrication of the PI- and metal thin-film-based substrate. Application of the PI in its liquid form by spin coating and annealing directly on dice led to a strong (59.1 ± 13.7 MPa^44^, comparable with die fixation by polymeric adhesive) and void-free contact between dice and substrate without additional adhesion promoters. This enabled a thickness of the substrate in the lower micrometer range, which is a prerequisite for conformable PI substrates. The mean translation was as low as 5.13 ± 3.06 µm (*n* = 90) (in-plane) and 4.26 ± 2.53 µm (*n* = 45) (out-of-plane), and a mean rotation down to 0.80 ± 0.49° (*n* = 45) (in-plane) and 0.90 ± 0.50° (*n* = 45) (out-of-plane) was achieved for dice of 390 µm edge length. Handling thin and fragile dice was circumvented by grinding dice to the desired thickness at the end of the process sequence (minimum thickness of 24 µm).

## Conclusion

Hybrid bioelectronic devices, consisting of sophisticated ICs modularly distributed on miniaturized flexible polymeric substrates, are a promising approach to overcome a central challenge in neural implants: the combination of complex functionality at high channel density and longevity in a biological environment. The virtue of this method is the possibility of scaling down the size of Si-based dice and distributing loads between dice connected by thin-film interconnect lines on the flexible substrate. This is accompanied by a lower bending stiffness compared to a single but larger die while maintaining good adaptability of the technical system in terms of the range of function, substrate dimensions, and target anatomy. In this study, a novel microfabrication procedure was developed where multiple dice can be batch-transferred into mechanically flexible PI substrates and interconnected with great precision on the wafer level. The process allows for dice of arbitrary shapes, sizes, and positions on the PI substrate. Handling of thin and fragile dice is avoided since grinding to the desired thickness is conducted after any handling, transfer, embedding, and interconnecting. It has thus been defined how ASICs can be integrated into mechanically compliant PI-based substrates, taking into account the previously given requirements. The next steps should be to (1) test the function of the system acutely in vivo, (2) verify the stability in animal models, and (3) validate the assumptions on structural biocompatibility. The conclusions drawn in this work and the processes developed herein lay the foundation for further development in next-generation neural implants that allow adaptation to a wide range of anatomical boundary conditions and may offer significant value for preclinical studies on the long-term application of hybrid implants.

## Methods

### Design assessment: implant geometry

Simulations were performed with COMSOL Multiphysics 5.3 (COMSOL Inc., Sweden) under the solid mechanics module and studied under stationary conditions. The deformation of a rat’s cortex was modeled thoroughly in ref. ^19^ with validation in vivo. This existing model was used as a starting point for the approximation of the mechanical interaction between the implant (substrate was modeled as a rectangle with a length of 2 mm and thickness of 10 µm) and cortex, which was 2D approximated as a quarter circle (mirrored at x = 0) with a radius of 3 mm (a radius found in the somatosensory cortex of rats^32^). Both bodies were assumed to be linearly elastic with Young’s modulus *E*, Poisson’s ratio *v*, and density *ρ* (Table 1^19,45^). Deformation of the PI substrate due to surface tension and capillary forces was approximated as a point load of 0.21 mN applied at the outer lower edge of the substrate, perpendicular to the tangent of the deformed geometry. Domains were connected through triangular elements, which were refined gradually from 80 µm to an element size of 4 µm on the cortex surface with a conformability threshold set to 90% of the array’s length in contact with the cortex. Cortex depression and curvature along the surface and cortex shape after loading were studied.

**Table 1:** Mechanical properties of polyimide, silicon, and rat brain used for modeling (Young’s modulus *E*, Poisson’s ratio *v*, density *ρ*)

Material	*E* in Pa	*ν*	*ρ* in kg/m3
Polyimide	9 ∙ 10^9^	0.34	1100
Silicon	170 ∙ 10^9^	0.28	2329
Brain cortex	1.75 ∙ 10^3^	0.40	1027

**Fig. 5:**
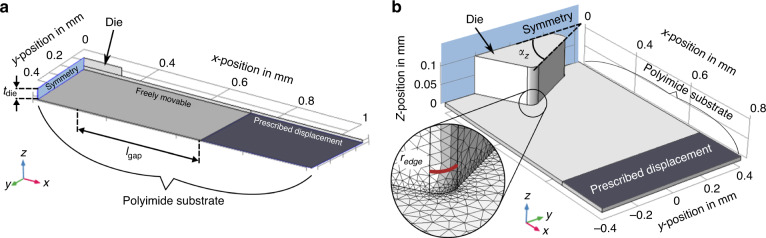
Graphic representation of individual domains defined in the finite element models. **a** Visualization of the physical boundary condition of the prescribed displacement of the polyimide (PI) substrate (dark gray), fixation of the object, symmetry plane (light blue), and freely movable domain to determine the deformation of a quadratic die (edge length *l*_die_ of 400 µm) on a PI substrate caused by conformation to a cylindrical surface. **b** Visualization of model to determine mechanical stress concerning the variation of edge radius fillet *r*_edge_ and rotational misalignment by an angle *α*_Z_ around the die center in the *XY*-plane. The inset shows an enlarged view of the refined mesh at the corners and edges of the contact surface between the die and PI substrate

A die was modeled with corners filleted by a radius of curvature *r*_edge_ (varied between 20 and 50 µm) on a PI substrate, and rotation of an angle *α*_Z_ around the die center in the *XY*-plane was varied between 0 and 45° (Fig. 5b). The PI substrate was deformed, and the implant was dimensioned as follows: *l*_die_ = 400 µm, *t*_die_ = 100 µm, *t*_PI_ = 10 µm, gap length = 415 µm. The substrate and die were connected as an assembly in COMSOL, the adhesive strength was set to 58 MPa (according to experimental shear-test data^44^), and the von Mises stress on the interface between the substrate and die was investigated in a parametric study. A load of 50 Pa was applied on the entire substrate bottom surface to account for deformation due to the surface tension of the cerebrospinal fluid alongside the substrate^19^.

### Transfer technology

The transfer process was based on placing individual dice into cavities in a carrier, which allowed further leveled standard microfabrication steps, e.g., photolithography, dry etching, physical vapor deposition (PVD), and mechanical grinding. Precise positioning was guaranteed, and setting the relative contact pad position on all dice prevented contact shorting or pads not connected by interconnects deposited in batch. Handling of ultrathin and fragile dice was averted by thinning at the end of the process sequence.

**Fig. 6:**
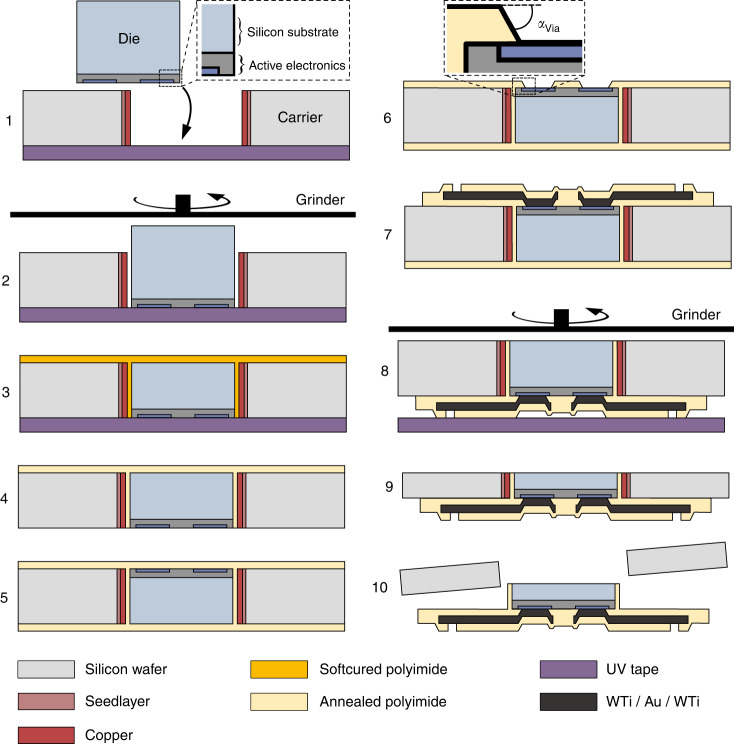
**Simplified sketch of the process sequence for batch transfer of silicon-based dice into flexible polyimide (PI) and metal thin-film-based neural implants.** **1** Die placement in the carrier. **2** Backside leveling. **3** Soft-curing of the PI backside fixture and removal of grinding tape. **4** Annealing of the PI in nitrogen. **5** PI substrate based on frontside. **6** Opening of PI over die contact pads beveled sidewalls by reactive ion etching (RIE). **7** Sputter deposition of metallization, application of PI topside substrate, and dry etching of device outlines and openings for contact pads. **8** Backside thinning of dice and carriers. **9** Removal of grinding tape. **10** Release of PI-based structures by dissolving the sacrificial layer

For carrier fabrication, a 4-inch Si wafer with a thickness of 525 µm, low bow (<20 µm), and low warp (<20 µm) was used. Cavities were dry-etched through the wafer by deep reactive ion etching (DRIE, alternating SF_6_ etching, and C_4_F_8_ passivation) (SPTS Technologies, United Kingdom) using a photoresist mask (26 µm AZ 10XT, MicroChemicals GmbH, Germany). Next, a gold (Au) seed layer for galvanic deposition of Cu was applied from the frontside under carrier rotation. To ensure that galvanic deposition (and therefore an increase in surface roughness) only takes place on the cavity sidewalls, the seed layer on the carrier frontside was protected by dicing tape (SWT 10+R, Nitto Denko, Japan). Subsequently, 6 µm of Cu was deposited, which was chosen as a sacrificial layer since it could be selectively etched against other materials employed to facilitate die release after device finalization. After the mechanical removal of the tape, the seed layer was removed from the frontside by reactive ions etching (RIE) was performed in argon plasma at 200 W (SPTS Technologies, United Kingdom). UV-light-curable back grinding tape (E3125 KL, LINTEC Corporation Advanced Materials Operations, Japan) was mounted on the frontside to fixate dice throughout the following process steps. Dice were placed inside the cavities with their active side (with connection pads) fixated by the tape on the carrier frontside (Fig. 6(1)). Subsequently, the backside of the carrier and dice were leveled with an automatic surface grinder (Fig. 6(2)) (DAG810, Disco Corporation, Japan). On the leveled backside, a 4-µm-thin PI (BPDA-PPD) layer was applied (Fig. 6(3)). Spin coating of PI was performed with a 2-min hold period before and after rotation to allow the PI to level and cover height topographies on the wafer. To exclude the grinding tape from the temperature impact of the annealing process, it was exposed to UV light (RAD 2000, LINTEC Corporation Advanced Materials Operations, Japan) and peeled off after PI soft-curing at 90 °C for 180 s on a hotplate. Thus, the PI backside layer replaced the tape as die fixation throughout the following process steps. Finally, the annealing of PI on the backside was performed at 450 °C for 10 min (YES PB HV, Yield Engineering Systems, USA) in a nitrogen atmosphere (Fig. 6(4)). On the frontside, 4 µm of PI was applied as the implant base layer (Fig. 6(5)) and opened above the die contact pads by RIE at 100 W in oxygen plasma (Fig. 6(6)) using a photoresist mask (AZ 10XT). To facilitate a uniform PVD coating from the PI base layer surface down to the die contacts, resist underexposure was performed to transfer beveled sidewalls into the PI openings. After resist stripping, sputter deposition of the metal layer stack (50 nm/300 nm/50 nm) of tungsten titanium (WTi), Au and WTi was performed (Star 100 PentaCo, FHR Anlagenbau GmbH, Germany) using 4 µm of negative tone photoresist ma-N-1440 (micro resist technology GmbH, Germany) and the lift-off technique to remove excess material. The PI surface was activated in oxygen plasma in an RIE machine for 45 s at 80 W. Oxygen plasma surface activation was performed to enhance adhesion between the PI base and the hereafter applied PI top layer (4 µm), which was opened by RIE above the contact pads and between individual devices using a photoresist mask (AZ 10XT). At this point, device structuring was complete (Fig. 6(7)), and RIE at 100 W could remove the PI backside layer in oxygen plasma. Afterward, the dice were thinned together with the carrier from the backside to the desired thickness (Fig. 6(8)) and cleaned. In correspondence with the FEM simulation, grinding was carried out down to 100 µm; however, to validate the transfer process at the lower extreme value of the die thickness, grinding to 25 µm (thickness of the active electronics) was also tested. After exposure, the carrier was removed from the UV-curable tape (Fig. 6(9)), and finally, the devices were released by dissolving the Cu sacrificial layer selectively with ammonium persulfate solution and rinsed with deionized water (Fig. 6(10)). Focused ion beam (FIB) cutting and scanning electron microscopy (SEM) of surfaces and cross-sections was performed in a Scios 2 HiVac (Thermo Fisher Scientific, Waltham, MA, USA). The adhesion performance of transferred dice on the target substrate was characterized with shear tests on a DAGE4000 Multipurpose Bondtester (Nordson Dage, United Kingdom) with a DS100 cartridge, a test speed of 200 µm/s and a shear height of 50 µm. The substrate holder was aligned to the shearing tool with an integrated microscope so that the dice to be sheared were parallel to the travel direction. A more detailed description of the methods is presented elsewhere^44^. The transition from the thin-film metal interconnects to the dice contact pads was characterized by 4‑wire resistance measurements with a multimeter (Agilent 34410 A, Keysight, USA) and a needle prober (Süss MicroTec SE, Germany). The measured resistance includes the resistance of the interconnect line, the metal structure on the test dice, and the contact resistance between both. The electrical resistance of test dice metal structures was measured on nontransferred dice with the same method as a comparison.

### Accuracy assessment: die placement

**Fig. 7:**
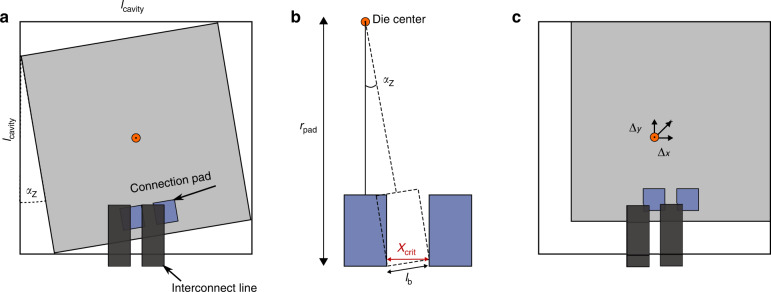
**Assessment of critical positioning errors**. Schematic representation of **a** a die rotated by *α*_Z_, which leads to an electrical shortage of two contact pads by an interconnect line, **b** location relations with the dashed line indicating the position of the contact pad after rotation, and **c** translation error leading to an electrical shortage of two contact pads by an interconnect line

Assessments of possible die rotation with given process parameters were performed, which can be used to optimize die design in terms of edge, size, and shape of contact pads. The risk of misalignment between different layers of the device during fabrication must be minimized to prevent possible misconnection. Figure 7a sketches a die and two contact pads with an outer edge at a distance *r*_pad_ from the center of the die (Fig. 7b). The die was placed in a cavity of length *l*_cavity_ and rotated by an angle *α*_Z_ from the ideal orientation with respect to the cavity sidewall.

The critical angle *α*_Z,crit_, which would lead to a misalignment causing a shortage of contact pads by interconnects, was calculated as follows: *x*_crit_ was the deviation in the *X*-direction caused by rotation that would lead to contact pads being shortened by an interconnect. Under the assumption of small angles, this distance could be approximated by the base length of the isosceles triangle *l*_b_, spanned by rotation of *r*_pad_ around *α*_Z_ (Eq. (1)). Solving for *α*_Z_ and applying the critical distance leads to the critical rotation angle *α*_Z,crit_ (Eq. (2)). The critical in-plane translation could be extracted as the distance between pads (Fig. 7c).

1 $$l_{\rm{b}} = 2 \cdot r_{\rm{pad}} \cdot \sin \left( {\frac{{\alpha _{\rm{Z}}}}{2}} \right)$$

2 $$\alpha _{{{\rm{Z}}},{{{\mathrm{crit}}}}} = 2 \cdot \sin ^{ - 1}\left( {\frac{{x_{{{{\mathrm{crit}}}}}}}{{2 \cdot r_{{{{\mathrm{pad}}}}}}}} \right)$$

The in-plane rotational error *α*_Z_ and translational errors *Δx* and *Δy* of dice were analyzed with optical micrographs and ImageJ software (ImageJ.net, Wayne Rasband, USA) for different cavity lengths *l*_cavity_. The die center deviation in the *Z*-direction from the ideal position in the *XY*-plane *Δz* and the die tilt *α*_X_ and *α*_Y_ around the *X*- and *Y*-axes were extracted from topography scans with a mechanical profilometer (Dektak 150, Veeco Instruments Inc., USA).
